# From admission to discharge: a systematic review of clinical natural language processing along the patient journey

**DOI:** 10.1186/s12911-024-02641-w

**Published:** 2024-08-29

**Authors:** Katrin Klug, Katharina Beckh, Dario Antweiler, Nilesh Chakraborty, Giulia Baldini, Katharina Laue, René Hosch, Felix Nensa, Martin Schuler, Sven Giesselbach

**Affiliations:** 1 Fraunhofer IAIS, Sankt Augustin, Germany; 2 Institute of Interventional and Diagnostic Radiology and Neuroradiology, University Hospital Essen, Essen, Germany; 3 Institute for Artificial Intelligence in Medicine, University Hospital Essen, Essen, Germany; 4 West German Cancer Centre, University Hospital Essen, Essen, Germany

**Keywords:** Clinical natural language processing, Patient journey, Out-of-distribution generalization, Explainable ML, Bias

## Abstract

**Background:**

Medical text, as part of an electronic health record, is an essential information source in healthcare. Although natural language processing (NLP) techniques for medical text are developing fast, successful transfer into clinical practice has been rare. Especially the hospital domain offers great potential while facing several challenges including many documents per patient, multiple departments and complex interrelated processes.

**Methods:**

In this work, we survey relevant literature to identify and classify approaches which exploit NLP in the clinical context. Our contribution involves a systematic mapping of related research onto a prototypical patient journey in the hospital, along which medical documents are created, processed and consumed by hospital staff and patients themselves. Specifically, we reviewed which dataset types, dataset languages, model architectures and tasks are researched in current clinical NLP research. Additionally, we extract and analyze major obstacles during development and implementation. We discuss options to address them and argue for a focus on bias mitigation and model explainability.

**Results:**

While a patient’s hospital journey produces a significant amount of structured and unstructured documents, certain steps and documents receive more research attention than others. Diagnosis, Admission and Discharge are clinical patient steps that are researched often across the surveyed paper. In contrast, our findings reveal significant under-researched areas such as Treatment, Billing, After Care, and Smart Home. Leveraging NLP in these stages can greatly enhance clinical decision-making and patient outcomes. Additionally, clinical NLP models are mostly based on radiology reports, discharge letters and admission notes, even though we have shown that many other documents are produced throughout the patient journey. There is a significant opportunity in analyzing a wider range of medical documents produced throughout the patient journey to improve the applicability and impact of NLP in healthcare.

**Conclusions:**

Our findings suggest that there is a significant opportunity to leverage NLP approaches to advance clinical decision-making systems, as there remains a considerable understudied potential for the analysis of patient journey data.

## Introduction

Natural language processing (NLP) has achieved significant success in applications such as translation, speech recognition, text generation, virtual assistants, and chatbots [1, 2]. These applications cover industrial, creative as well as lifestyle domains, and more recently, also the healthcare sector [3, 4]. Due to an increasing number of patients, rising costs and larger amounts of data, there is a high demand for automated processing of health-related documents. Hospitals struggle to provide high-quality care due to the complexity of patient histories and the high volume of medical documents generated during hospital stays, including reports from pathology, radiology, laboratory, surgery, and care documentation [5]. This information and is crucial for any decision on diagnostics, therapy or subsequent care. Significant effort is dedicated to the tasks of writing, filing, sorting, searching, retrieving, issuing, and managing medical records by the clinicians. But it is nearly impossible for clinicians to process this bulk of information [5]. Therefore, it is highly desirable to supply healthcare professionals as well as patients with information contained in these full texts by extracting data, mapping it onto clinical guidelines or otherwise inform their decisions. Hence, almost all Clinical Decision Support Systems (CDSS) depend on a continuous and reliable processing of clinical text [6]. Despite the promising capabilities of NLP for enhancing clinical decision-making and operational efficiency, its integration into real-world healthcare settings remains limited due to challenges such as data quality, lack of standardization, and inadequate alignment with clinical workflows [7]. This study aims to address these challenges and provide solutions to facilitate the integration of NLP in clinical environments. The significance of this research lies in its potential to bridge the gap between NLP research and its application, ultimately contributing to improved patient outcomes and operational efficiency. Our goal is to equip researchers with established and successful approaches for clinical NLP. Together with the mounting number of publications in this research area, this motivates a systematic survey of existing approaches.

In this survey, we report on the current state of research in clinical NLP along the different stages of a patient’s journey through a hospital. In collaboration with doctors as domain experts, we have created a prototypical patient journey. In total, we reviewed 8.527 papers, applying a filtering and screening process to include medical and clinical papers. On the one hand, we used NLP-related tags to map the papers to relevant NLP tasks, models, datasets, and data languages. On the other hand, we used clinical tags, such as general patient journey and patient journey documents, to ensure mapping the NLP applications to the actual patient journey. Previous work, such as that by [7], provides a foundation for understanding the practical considerations necessary for developing effective clinical NLP systems.

We identify gaps between research and clinical application of NLP in hospitals, as well as areas that require further exploration and development. In particular, our results show that there is a lack of research in developing trustworthy models, and we thus highlight distinct challenges in this field of NLP in the clinical setting and suggest an outline on how to address them during development.

We begin by describing related work in the area of NLP for hospital documents. The subsequent section describes in detail a prototypical patient journey, along which medical documents are created, processed and consumed by hospital staff and patients themselves. We describe our methodology of identification, selection and extraction of relevant publications in the literature and the key insights obtained. In the main section, we map recognized concepts onto our framework consisting of multiple technical and medical dimensions and follow up with an analysis and discussion of the results. The final section concludes with a description of overarching patterns and suggestions for the applications of NLP systems in clinical practice.

## Related work

The use of NLP in medicine has been the focus of several surveys in recent years. Topics that have been investigated include deep learning architectures deployed in medical imaging and NLP [8], or the implementation of task-oriented dialogue systems for healthcare applications [9]. Other studies have concentrated on NLP systems for capturing and standardizing unstructured clinical information and generate structured data [10]. Most of the mentioned surveys on NLP in the medical domain focus on a specific task, such as converting image to text or dialogue systems, and do not provide a holistic view of NLP applications in healthcare.

Recently, some studies have explored the patient journey in the hospital. While [11] applied process mining techniques to the patient journey to improve the patients’ satisfaction, [12] discussed general AI opportunities along the patient journey. To the best of our knowledge, no prior research has focused on mapping the patient journey onto NLP tasks in research. Therefore, in our survey, we concentrate on this mapping to analyze the current NLP research and applications along the patient journey by reviewing relevant publications. Unlike previous studies that focus on specific tasks, our review provides a holistic view of NLP applications throughout the patient journey, identifying gaps in areas such as After Care and Smart Home. Our approach integrates NLP into various stages of the patient journey, offering a detailed perspective that previous studies lack. By mapping the patient journey onto NLP tasks, we provide insights into how NLP can be utilized not only in clinical settings but also in post-discharge and home care scenarios. This broadens the scope of NLP applications beyond traditional settings. Furthermore, our approach identifies overlooked areas, offering a roadmap for future research and development in NLP applications across patient care.

## Patient journey

To better illustrate the amount of unstructured documents that patients encounter during their hospital stay, we employed a case study approach to present the hospital journey of a cancer patient. Specifically, we focused on the patient journey of a lung cancer patient, as it is one of the most commonly diagnosed subtypes of cancer, and cancer is the second leading cause of death in the western world [13].

**Fig. 1:**
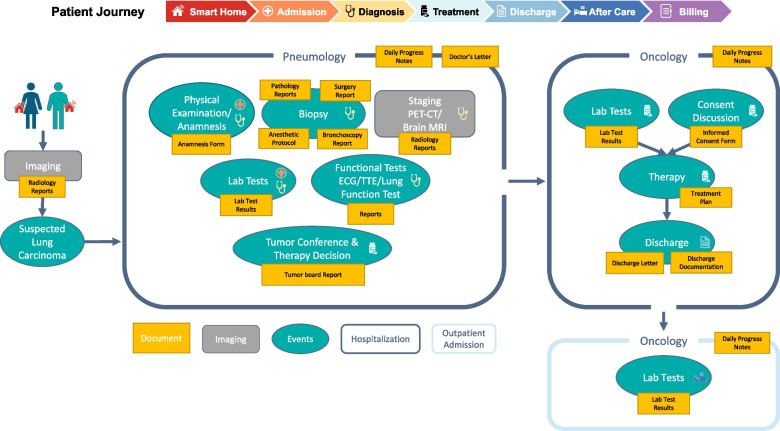
Typically, a suspicion of lung carcinoma leads to admission to the pneumology department. Multiple tests are conducted to reach a diagnosis. The treatment options for the patient are discussed by a multidisciplinary tumor board and the patient is transferred to the oncology department to undergo the chosen therapy. Once completed, the patient is discharged from the hospital, but may continue to visit for follow-up checks to ensure effective treatment. Documents collected during this journey are highlighted in yellow. The steps in this example are marked with the symbols of the corresponding phases of the general patient journey, shown at the top

The patient journey begins with a suspected diagnosis of lung cancer, followed by complex diagnostic procedures and resulting in cancer treatment [14, 15], as shown in Fig. 1, where we also highlighted the emerging documents during this process. Medical information systems typically document these findings and information in unstructured text, except for laboratory test results, which are usually available in a structured format.

Most commonly, lung cancer is suspected based on arising symptoms or as an incidental finding in an imaging study. In the next step, the patient gets hospitalized for further diagnostic procedures, usually in the pneumology department. This diagnostic pathway starts with the anamnesis and a physical examination by the physician as well as laboratory tests, followed by a tumor biopsy and a lymph node sampling for histological examination and staging. The tumor staging is completed by performing further imaging studies. For the staging of lung cancer, a positron emission tomography-computed tomography (PET-CT) and a brain magnetic resonance imaging (MRI) are the gold standard. To evaluate the cardiopulmonary function of the patient, functional tests by means of electrocardiogram (ECG), transthoracic echocardiogram (TTE) and pulmonary function tests are carried out. Each of these steps produces one or multiple reports. When all the diagnostic information is available, the treatment strategies are discussed in a multidisciplinary lung cancer tumor board consisting of medical oncologists, radiation oncologists, pulmonologists, thoracic surgeons, radiologists and/or nuclear medicine specialists and pathologists. If a chemotherapy is recommended, the patient is transferred to the oncology department. Once the consent discussion has been completed, a systemic anticancer therapy such as a chemotherapy and/or an immunotherapy is applied. Following the systemic therapy, the patient gets discharged. Approximately one week after the application of the therapy, an ambulant laboratory test is recommended. If necessary, the patient returns for the second cycle of chemotherapy after a typical waiting period of two to three weeks.

From this use case we initially identified five main patient journey stages: **Admission**, **Diagnosis**, **Treatment**, **Discharge** and **After Care**. In each of these stages, different documents are collected, processed and used by other clinicians in later stages. As shown in Fig. 1, the corresponding events for each stage have been mapped using respective symbols. The diagnostic phase is typically the most document-intensive, as patients undergo numerous tests and procedures to obtain a suitable diagnosis. The treatment phase also generates a significant amount of documentation, owing to the close monitoring of the patient’s progress to ensure that the treatment is proceeding smoothly. Our particular patient journey involves fewer documents in the other three stages. Although not directly evident in the patient journey, we have included the initial stage of the journey, **Smart Home**, as Internet of Things (IoT) applications are becoming increasingly relevant in the healthcare sector [16]. Patients may, for example, bring heart rate measurements monitored using their smartwatches, which could be used as an additional diagnostic tool. Another part of the journey that does not directly influence the patient care is **Billing**, which is a source of multiple unstructured documents.

In summary, the hospital journey of a patient, in this example a cancer patient, generates a significant amount of structured and unstructured documents. To better understand this process, we have divided the journey into seven main stages, where each stage produces different types of documents that are crucial to the overall care of the patient. By recognizing the document-intensive nature of the patient journey and the potential for unstructured data to impede care, we can begin to explore the benefits of implementing NLP technologies to streamline document handling and improve patient outcomes.

## Methods

In order to analyze the transfer of NLP research into the clinical domain and map the actual use of NLP throughout the patient journey, we conducted a systematic review of 8.527 papers based on publication venue, date, and title combined with a keyword search as our selection criteria. The tagging was performed for two dimensions. The first dimension concentrated on NLP-related tags to map the papers to relevant NLP tasks, models, datasets, and data languages. The second dimension focused on clinical tags, such as general patient journey and patient journey documents. The final list of publications was then screened with NLP-related and patient journey related tags. A team of four reviewers annotated the papers, and the papers were equally split among the reviewers. Each paper was annotated by two reviewers and in case of doubts, a third reviewer was used for tie-breaks. In visualizing the results, we employed Python along with its packages including Seaborn, Matplotlib, Pandas, Plotly, and Sankey, ensuring comprehensive data representation. In the following, we provide an overview of the methodology used in our review.

### Search criteria and screening process

In the following, we describe our search criteria and screening process for selecting literature.

**Publication venue**. In our systematic review we focused on articles published in NLP conferences from the ACL anthology (ACL, EMNLP, COLING, CoNLL, EACL, NAACL, AACL) and workshops from end of 2018 to December 2022. Specifically, we targeted workshops that have a medical research focus, like BioNLP, NLPMC, SMM4H, ClinicalNLP, LOUHI. All articles were last extracted in January 2023.

**Title screening**. To further refine the search, we employed a keyword filtering process. We selected relevant keywords through discussions with healthcare professionals in the clinical domain and screened the titles of the initial list of papers. The following list of keywords was used: *medical, medicine, health, care, patient, treat, cancer, hospital, surgery, surgical, drug, emergency, doctor, surgeon, human, disease, diagnosis, trauma, report, discharge, clinical*.

**Abstract and paper screening**.

*Relevance and Medical Domain:* Next, we filtered our remaining paper list by screening the abstracts and excluding papers that are not relevant for the medical domain. Additionally, we excluded papers that were research or tutorial proposals, or demo papers.

**Fig. 2:**
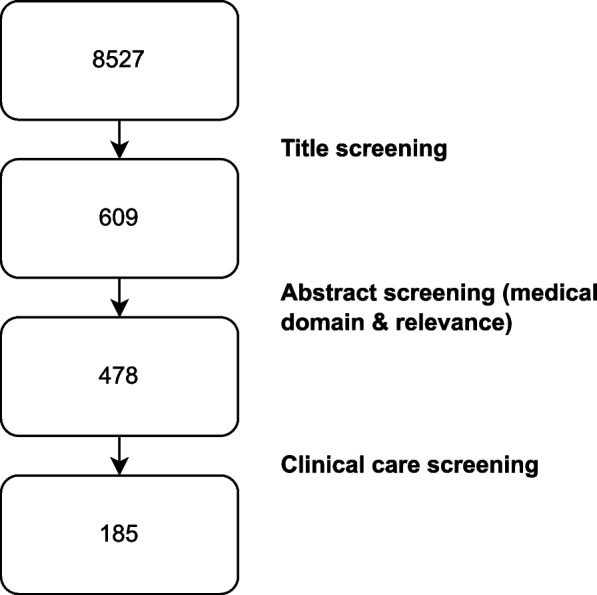
Amount of papers per screening process step for the selection of the reviewed paper list

*Clinical Screening:* As our research focuses on clinical NLP and the patient journey in a hospital, we further refined the list by identifying the papers that are relevant for the clinical domain. Research analyzing bio markers or social media posts were excluded by our clinical screening process. The initial collection, based on the selection of the publication venue and the years, consisted of 8.527 papers. The filtering process led to 609 publications after the keyword search in our title screening, 478 after relevance and medical domain filtering and remaining 185 clinical domain papers (see Fig. 2).

### Tagging process

Our review involved mapping every paper of our screening process to several NLP-related tags, with the aim of identifying which models, tasks, datasets, and data languages are most commonly used in healthcare NLP research. To identify the current applications of NLP research in the hospital, we included tags for patient journey and document types. Specifically, we assigned tags to each paper based on the stage of the patient journey that was being addressed (e.g., diagnosis, treatment, admission), as well as the type of patient journey document that was being analyzed (e.g., clinical notes, discharge summaries, radiology reports) (see “Patient journey” section). For this part of the analysis, we only focused on the papers left after the Clinical Screening process, as our patient journey concentrates on a hospital patient (see “Search criteria and screening process” section). We assigned multiple tags where applicable, e.g. when multiple datasets were used. Our detailed tagging scheme can be seen in Tables 1 and 2.

**Table 1:** Patient journey tags

Category	Tags
General patient journey	P1: Smart Home (e.g. preclinical data, home-monitoring devices)
	P2: Admission
	P3: Diagnosis
	P4: Treatment
	P5: After Care
	P6: Discharge
	P7: Billing
	P8: Other
Patient journey report type	R0: Admission notes
	R1: Radiology report
	R2: Discharge letter
	R3: Documented histories
	R4: Pathology report
	R5: Tumour conference decisions
	R6: Lab results
	R7: Surgery report
	R8: Other

**Table 2:** General NLP-related tags

Category	Tags
Data type	D1: All patient related records
	D2: Clinical studies
	D3: Registry data
	D4: Protein data
	D5: Genome data
	D6: Forum posts, chatlogs, social media
	D7: Speech data, dialogue data
	D8: Image data
	D9: Knowledge graph, thesaurus
	D10: Medical online information (Wikipedia, drug information, FAQs, etc.)
	D11: Patents
	D12: News articles and press releases
	D13: Clinical guidelines
Data language	free text, e.g. English, German
Task	T1: Classification
	T2: Information extraction
	T3: Clustering
	T4: Text generation
	T5: Embeddings/representations
	T6: New dataset creation
	T7: Question answering
	T8: Text summarization
	T9: Translation
	T10: Reinforcement learning
	T11: Recommender system
	T12: Natural Language Inference and entailment
	T13: Topic model
	T14: Probing
	T15: Ranking
Secondary task	S1: Explainability
	S2: Domain adaptation
	S3: Bias, fairness
	S4: Resource-awareness
Model type	M1: Transformer-variants (BERT, RoBERTa etc.)
	M2: Convolutional Neural Nets (CNNs)
	M3: Recurrent Neural Nets (RNN, LSTM)
	M4: Statistical models (Bayes, conditional probabilities, CRF)
	M5: Graph Neural Networks (GNNs)
	M6: Dimension reduction
	M7: Graphical models (PGM)
	M8: Generative Adversarial Networks (GANs)
	M9: Rule-based models
	M10: Decision trees, Random Forest
	M11: Support Vector Machines (SVM)
	M12: K-nearest neighbors (kNN)
	M13: Pointer generator model
	M14: Feedforward neural network
	M15: Logistic regression
	M16: Linear regression
	No contribution

**Fig. 3:**
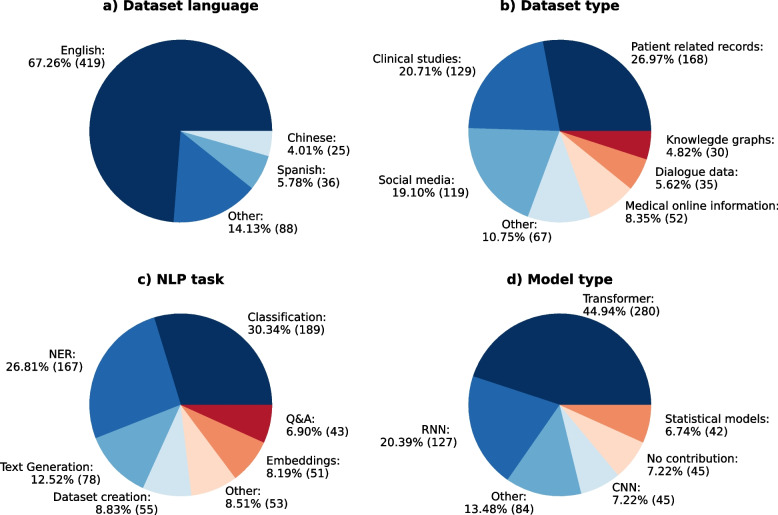
Distribution of the number of papers per NLP related tag category: (**a**) dataset language (**b**) dataset type (**c**) NLP task (**d**) model type.Values that fell below the 5% threshold were aggregated into “Other” category for the purposes of analysis, except for dataset language, where we display the top three dataset languages

## Results

In this section, we present our findings in relation to (1) NLP systems in the healthcare domain and (2) along the patient journey.

### Mapping NLP tags

In the following, we describe the results of our review with respect to the NLP methodologies and datasets implemented in the healthcare domain.

**Dataset language**: Various studies have analyzed or explored datasets consisting of multiple data languages. Through the analysis of 487 papers, we observed that English was the most frequently used dataset language (419). The second and third most used dataset language were Spanish (36) and Chinese (25). The remaining 237 languages were classified under the ‘Other’ category (see Fig. 3).

**Dataset type**: In terms of datasets, we found that patient related data, like electronic health records, were the most commonly used sources of data (27%), followed by clinical studies (20.7%), and forum posts, chat logs, social media datasets (19.1%), as demonstrated in Fig. 3.

**Fig. 4:**
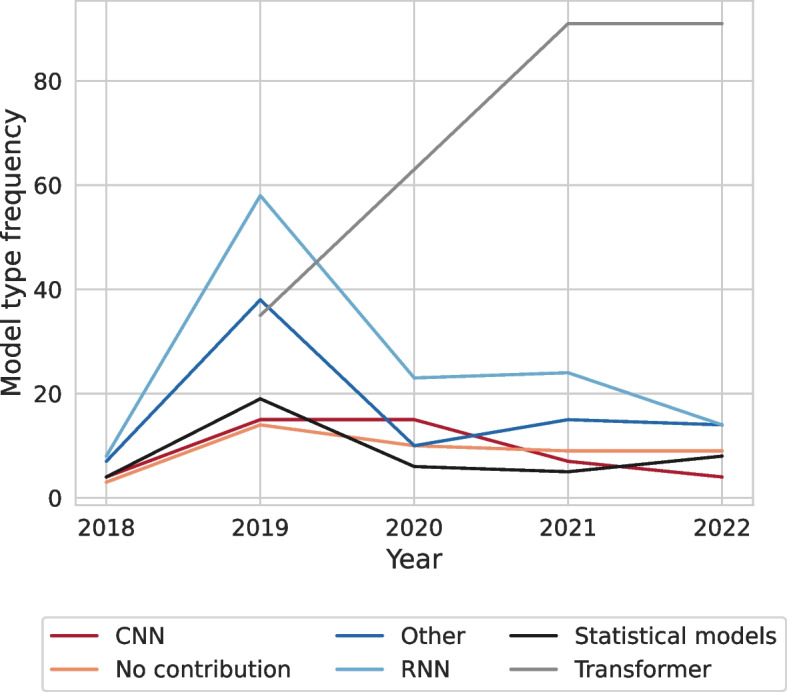
Development of model types used in NLP research over the past years

**Model type**: Figure 3 displays that transformer-based models were the most commonly used type of NLP model across a variety of tasks (44.94%), followed by recurrent neural networks (RNN) (20.39%). As shown in Fig. 4, in 2019, RNNs were still used more frequently than transformer-based models. The use of transformer-based models increased over a four-year period, culminating in a peak in 2021 and 2022.

**NLP Task**: Finally, we observed that certain tasks, such as classification with almost 30%, information extraction with 26.81% and text generation/text summarization which account for 12.52%, were more frequently studied than others.

### Mapping of patient journey

**Fig. 5:**
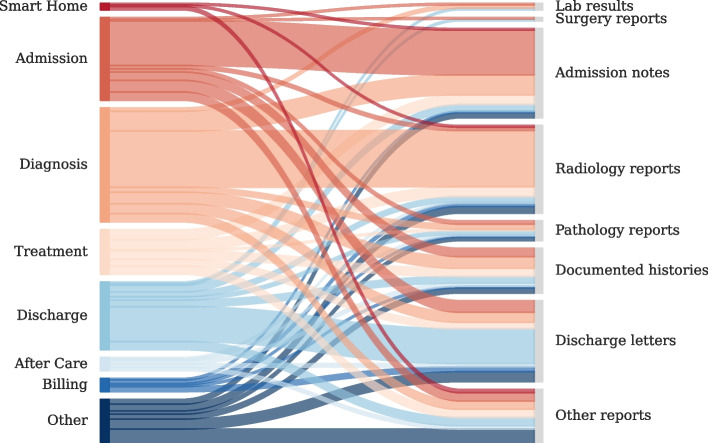
Patient Journey results: Comparison of patient journey steps (left side) with the patient journey documents (right side). The width of each stream shows how often the patient journey step or document type appeared in the reviewed papers

Analyzing the clinical patient journey, we observe that most of the clinical NLP papers focus on applications during the Diagnosis, Admission and Discharge phase of the patient, while referring to admission notes, radiology reports and discharge letters. It is remarkable that the most researched patient journey step is the Diagnosis while taking into account mostly radiology reports. As shown in Fig. 5, paper with the focus on the Treatment of the patients do not use a specific document type as a focal point, but an evenly distribution of admission notes, radiology reports, documented histories, discharge summaries and other document types. In contrast to that, patient journey steps like Smart Home, After Care, or Billing are less represented in the clinical NLP literature.

## Discussion

We observe that most of the publications in the medical NLP literature use English datasets, see also [17]. This indicates that other languages are under-researched in the medical domain whereby potential of clinical NLP application gets lost. Focusing on English data leads to an imbalance between non-English and English medical applications [18]. NLP models that are trained solely on English data may not perform as well when applied to other languages [19], because language models often rely on patterns and structures that are specific to a particular language, and these patterns may not be present in other languages [20]. Furthermore, by expanding the scope of research to other languages, researchers can uncover new patterns and structures that may not be present in English, leading to new breakthroughs and advancements in the field [19]. We already observe attempts to include non-English datasets. For example, most studies that dealt with Spanish datasets were published in the sixth and seventh Workshop on Social Media Mining for Health Applications and assessed Spanish tweets regarding health conditions [21, 22] or fifth Workshop on BioNLP Open Shared Tasks [23].

Looking at the data types, it is noteworthy that patient related records are used most frequently. Wornow et al. [17] found that there is an over-representation of models that were trained on the MIMIC-III dataset, as it is one of the few public available patient related datasets. Other publicly available datasets are needed to create models that are trained on larger clinical data with current knowledge about diseases and treatments [17]. Our findings show that current NLP research tends to focus on specific types of documents, such as radiology reports, discharge letters, and admission notes. There is a significant opportunity in analyzing a wider range of medical documents produced throughout the patient journey, such as care and disease progression documentation. Expanding the scope of analyzed documents to include a more diverse range of patient data will enhance the applicability and impact of NLP in healthcare. It is striking that although transformer models have been discussed in research since 2017 when they were first invented [24], they were mainly used in medical applications from 2020 onwards. This could indicate that there is a general delay in applying novel methods in the medical domain. While transformer models have shown promising results in research papers after 2017, implementing them in real-world applications may be challenging. Firstly, transformer models are often large and computationally intensive, which can make them difficult to run on resource-limited devices [25]. Additionally, transformer models require large amounts of high-quality training data. In the healthcare domain, obtaining such data can be difficult and limited due to sensitive patient related data that needs to be anonymized first [26–28]. Furthermore, transformer models are often referred to as “black boxes” because it can be difficult to understand how and why they predict a specific output. This lack of interpretability can make it challenging to use these models in the medical domain, where transparency and accountability are important [29–31].

Remarkable is the extent to which clinical systems can be supported by NLP technologies. We have shown that a patient generates a significant amount of structured and unstructured documents throughout the journey in a hospital. We observed that specific steps of the patient journey are researched more often than others. While Diagnosis and Admission are areas that are researched primarily in the clinical NLP community, there seem to be patient journey steps that are under-researched, e.g. Smart Home, Treatment, After Care, Billing, even though a lot of documents are produced for every patient in the hospital (see “Patient journey” section). In terms of documents, admission notes, radiology reports and discharge letters are used most frequently, which is in line with the previously analyzed patient journey steps (see Fig. 5). Patient journey steps such as Admission and Diagnosis are often considered to be critical in the patient journey, where early detection and intervention can have a significant impact on patient outcomes [32]. This may make them a priority for research and development of NLP models. One reason could again be that researchers may focus on points of the patient journey where high-quality data is available like admission notes, radiology and pathology reports. We observe that radiology reports are mainly used for the diagnosis in our review, which indicates that there is still a huge potential of clinical NLP technologies analyzing other report types than radiology reports for improving the diagnosis of a patient, as shown in Fig. 1. One reason could be that radiology and imaging reports are one part of the MIMIC-III dataset and predominantly used for research. Additionally, some data that is available and not related to patient data may be medically related but not clinically related. Examples are investigation of social media data to analyze the symptoms of COVID patients [33], detect patients’ emotional states [34] and mental illnesses [35, 36] or identify adverse drug reactions [37–40]. This type of data provides valuable insights into health trends and biological mechanisms, contributing to the broader understanding of medical science. However, it may not have immediate implications for patient care, unlike clinically related data, which includes patient history, diagnostic test results, and treatment outcomes. Our review shows that there is still a huge potential to support clinical decision systems with NLP methodologies, as the application opportunities lack behind the application reality. Researchers should explore NLP applications in Treatment and Billing phases to automate routine tasks, thereby reducing administrative burden and enhancing patient care which can lead to more accurate diagnoses and effective treatment plans. Practitioners can benefit from implementing NLP tools for better patient monitoring and follow-up in After Care. Furthermore, interdisciplinary collaboration between NLP researchers, clinicians, and healthcare administrators is crucial. Such collaborations ensure that NLP innovations are both technically sound and practically useful in clinical settings. Additionally, developing user-friendly NLP applications that are intuitive and easy to use can facilitate quicker adoption into clinical practice. By focusing on these aspects, both medical practitioners and researchers can use NLP methods to improve patient outcomes, streamline clinical workflows, and improve medical research.

### Challenges

There are several challenges which might prevent or slow down the process of applying NLP technologies in the hospital setting. While primary down-stream tasks can now be reasonably tackled, we are especially facing challenges in the field of trustworthiness. Contrary to our expectation, the reviewed papers largely omit this topic (ca. 16% of papers address trustworthy ML topics). In the following section we address this research opportunity and concentrate on the discussion of three challenges in the field: out-of-distribution generalization, explainability and bias.

#### Out-of-distribution generalization

One of the fundamental assumptions in supervised machine learning is the existence of identical and independently distributed data. Models perform well provided that test-time data points are distributed similarly to those used for training. In practice, we may have several sources of distribution shift between the training environment and the setting in which the model is deployed, leading to a lack of performance.

One of the sources of distribution shifts is subpopulation shift [41]. The training dataset may consist of data points that have the same label, while simultaneously having multiple distinct *subgroups* among them, i.e. the label only coarsely describes the meaningful variation within the population. A data subgroup might contain spurious correlations between its features and labels that do not hold outside this subgroup. If such subgroups are large enough, a model trained by minimizing empirical risk will latch onto these spurious correlations and underperform on “minority subgroups”. Shim et al. [42] investigate imbalance in a medical code prediction dataset in terms of demographic variables, and observe the issue of subpopulation shift while analyzing the performance differences of the model across demographic groups. This problem may be exacerbated when the model is tested in a deployment scenario with different distributions of demographic groups than that encountered during training. Holderness et al. [43] show that off-the-shelf sentiment classification models trained on general domain data do not perform very well on psychiatric patient health records. They further demonstrate that domain adaptation methods based on self-training and k-nearest neighbors can be used to adapt off-the-shelf models by leveraging a corpus of unlabeled electronic health record data.

Medical records which are written by clinicians from different specialties usually differ in terms of writing styles or terminologies used. In order to train Named Entity Recognition (NER) models on medical records, human-annotated datasets are needed. But the cost of human annotation makes it difficult to create labelled datasets in all specialties. Wang et al. [44] propose a label-aware domain transfer method for medical NER that learns a close feature mapping between source and target domains. This enables NER models trained on one specialty to be conveniently applied to another one with minimal annotation effort. Liu et al. [45] uses domain-adversarial training to learn whether a pair of disease phrases from different domains are semantically similar without requiring a lot of pairwise labelled data.

#### Explainable machine learning

One key challenge in adopting machine learning systems in the clinical domain is missing transparency [29–31]. NLP systems, in particular, suffer from opaqueness due to a reliance on deep neural networks. This is evident in the results of the literature analysis: Over 70% of papers rely on transformer variants, CNNs or RNNs, which are notoriously hard to interpret.

The field of explainable machine learning offers methods to address the lack of transparency [46–50]. Explainability in the clinical context is relevant for compliance legislation, system improvements and verification [51]. Explanations have different forms, such as text highlighting, rules or examples. The most prevalent explanation form in NLP is feature attribution, which typically highlights the features, e.g. tokens, that contribute most to a prediction [52]. While abundant explanation methods are available for predictive tasks, explanations for generative tasks are lacking and present an open research topic.

From the reviewed work, 21 papers (4%) explicitly mention explainability or interpretability. As is common in the NLP domain, the terms are mostly used interchangeably [53]. Roughly 40% use feature attribution as explanation form, which is in line with other reviews [52]. Another 40% integrate interpretable components or can be considered interpretable-by-design. Six papers report quantitative or qualitative evaluation, incl. three works which evaluate with one or two clinicians. In contrast, the majority of papers claims that the model is more interpretable or explainable without any quantification. Anecdotal evidence is common and a fundamental flaw in the field [54].

Ghassemi et al. [55] argue that current explainability methods are not sufficient for certain purposes in the clinical domain and argue to focus more strongly on validation practices. The explanation purpose is often not defined, which hinders the assessment of usefulness. In addition, we agree that rigorous validation is important and we start to see works in this direction, e.g. [56]. However, we emphasize that e.g. the robustness field is facing similar challenges with guarantees. A sole focus on validation is not sufficient to tackle transparency requirements. For this reason, we call for purpose-driven development and adequate evaluation to derive in which ways explanations are most beneficial for the clinical context.

#### Bias

In the medical domain, data bias is prevalent and imminent. While biomedical publications are mainly affected by reporting bias [57], medical record datasets can contain bias from multiple sources, including authorship, target audience, local practices, type of trigger, available time, deployed software or monetary incentives [58]. Whether employed dataset(s) are representative for a patient population is heavily dependent on data collection practices. For instance, in the case of acute kidney injury (AKI), less than 29% of all clinically identified AKI patients receive a corresponding ICD code in their patient record [59]. NLP models have trouble to differentiate sentences describing normalities from important abnormalities in radiology reports [60]. For machine learning systems that support clinical staff and patients in taking informed decisions, non-discrimination of protected groups is an essential goal. Handling bias in machine learning often consists of detecting and, when indicated, reducing bias. Detection is often driven by calculating statistical fairness metrics, such as *Group fairness* or *Equalized Odds*. It must be emphasized that no single metric is sufficient on its own, instead each application requires a combination of metrics, selected by a careful consideration of moral reasoning and domain-specific challenges [61]. Debiasing word embeddings and post-processing via equalized-odds can improve downstream clinical NLP tasks [62].

## Limitations

While we believe that our selection of NLP conferences provides valuable insights into current trends and advancements in the field, it is important to acknowledge the limitations of our methodology. Specifically, we chose to focus solely on NLP conferences from the ACL anthology and did not include general ML conferences or application-focused conferences from the medical domain in our analysis. This decision was made in order to provide a more focused and in-depth analysis of the technical aspects of the field. Future research may benefit from including a wider range of different NLP-related conferences and medical-related conferences in the analysis to better understand the intersection of technical advancements and real-world applications. While our study primarily focuses on mapping the patient journey onto NLP tasks, future work should expand on potential approaches to address bias mitigation and enhance model explainability. Addressing these challenges will further strengthen the deployment of NLP in healthcare, ensuring that the systems are fair, transparent and trustworthy.

## Conclusion

In this paper, we conducted a systematic literature review and mapped clinical NLP research onto a prototypical patient journey in the hospital. Specifically, we reviewed which dataset types, dataset languages, model architectures and tasks are researched in current clinical NLP research. Our results show that, while a patient’s hospital journey produces a significant amount of structured and unstructured documents, certain steps and documents receive more research attention than others. Diagnosis, Admission and Discharge are clinical patient steps that are researched often across the surveyed paper. In contrast, we found that Treatment, Billing, After Care, and Smart Home are under-researched. Additionally, clinical NLP models are mostly based on radiology reports, discharge letters and admission notes, even though we have shown that many other documents are produced throughout the patient journey. Our findings suggest that there is a significant opportunity to leverage NLP approaches to advance clinical decision-making systems, as there remains a considerable understudied potential for the analysis of patient journey data.

## Data Availability

The datasets used and/or analyzed during the current study are available from the corresponding author on reasonable request.

## Abbreviations

NLP: Natural Language Processing
CDSS: Clinical Decision Support Systems
PET-CT: Positron Emission Tomography-Computed Tomography
MRI: Magnetic Resonance Imaging
ECG: Electrocardiogram
TTE: Transthoracic Echocardiogram
IOT: Internet of Things
NER: Named Entity Recognition
CNN: Convolutional Neural Networks
RNN: Recurrent Neural Networks
AKI: Acute Kidney Injury
